# Characterization of the complete mitochondrial genome of *Cercospora nicotianae* (Ellis & Everh, 1893) (Dothideomycetes: Capnodiales)

**DOI:** 10.1080/23802359.2021.2013745

**Published:** 2021-12-28

**Authors:** Ning Lu, Xingjiang Chen, Zhongmei Pan, Liuti Cai, Yi Cao

**Affiliations:** a Plant Protection Division, Guizhou Academy of Tobacco Science, Guiyang, P. R. China; b College of Tobacco Science, Guizhou University, Guiyang, P. R. China

**Keywords:** *Cercospora nicotianae*, mitogenome, phylogenetic relationship

## Abstract

The mitochondrial genome of the fungal pathogen *Cercospora nicotianae* was sequenced for the first time using a combination of Illumina and Nanopore sequencing technologies. The circular genome is 27,737 bp in length with G + C content of 27.43%, consisting of 15 protein-coding genes, 26 transfer RNA genes and 2 ribosomal RNA genes. Phylogenetic analysis shows that the *C. nicotianae* mtDNA is closely related to *Pseudocercospora fijiensis*.

Frogeye leaf spot is one of the major diseases of tobacco (*Nicotiana tabacum*) infected by *Corynespora nicotianae* around the world, which causes great economic damages(Alasoadura and Fajola 1970; Huang and He 1994; Punit Kumar et al. 2016; Dixon et al. 2018; Guo 2021). The typical specimen was collected from tobacco planted in Shibing County of Guizhou Province, China (107.55 E; 27.08 N), and the strain was isolated and preserved in Guizhou Academy of Tobacco Science (specimen voucher: YC1110; Ning Lu; lu_ning@126.com). The total DNA was isolated using the DNeasy UltraClean Microbial Kits (QIAGEN, USA), and then respectively sequenced by Illumina (Novoseq 6000) and PromethION (Oxford Nanopore Technology, UK) after quality assessment. The results in quality control were subjected to subsequent assembly and annotation. MitoZ v2.3 completed the assembly process (Meng et al. 2019). MitoZ combined with the MFannot platform was used to run annotation to predict coding sequence (CDS), transfer RNA (tRNA), and ribosomal RNA (rRNA). A correction was then required according to the annotated results and the third-generation sequencing data. The phylogenetic trees were constructed using the MAFFT v7.487 (Katoh and Standley 2013) and FastTree 2.1.1 (Price et al. 2010). The mitogenome of the genus Corynespora was sequenced for the first time in this study.

The mitochondrial genome of *C. nicotianae* is a circular DNA molecule with 27,737 bp in length. The total nucleotide composition is A: 35.58%, T: 36.99%, G: 13.73%, and C: 13.70%. 15 protein-coding genes, 26 transfer RNA genes, and 2 ribosomal RNA genes were identified. The mitochondrial genome sequence was submitted to GenBank under accession number OK075294.

To identify the polygenetic position of *C. nicotianae*, a phylogenetic tree was generated based on 12 protein-coding genes shared by *C. nicotianae* and the mitochondrial genomes of 14 species from Dothideomycetes, which were downloaded from GenBank. The phylogeny analysis demonstrated that *C. nicotianae* of tobacco has a relatively close genetic relationship with *Pseudocercospora fijiensis* (Figure 1).

**Figure 1.**
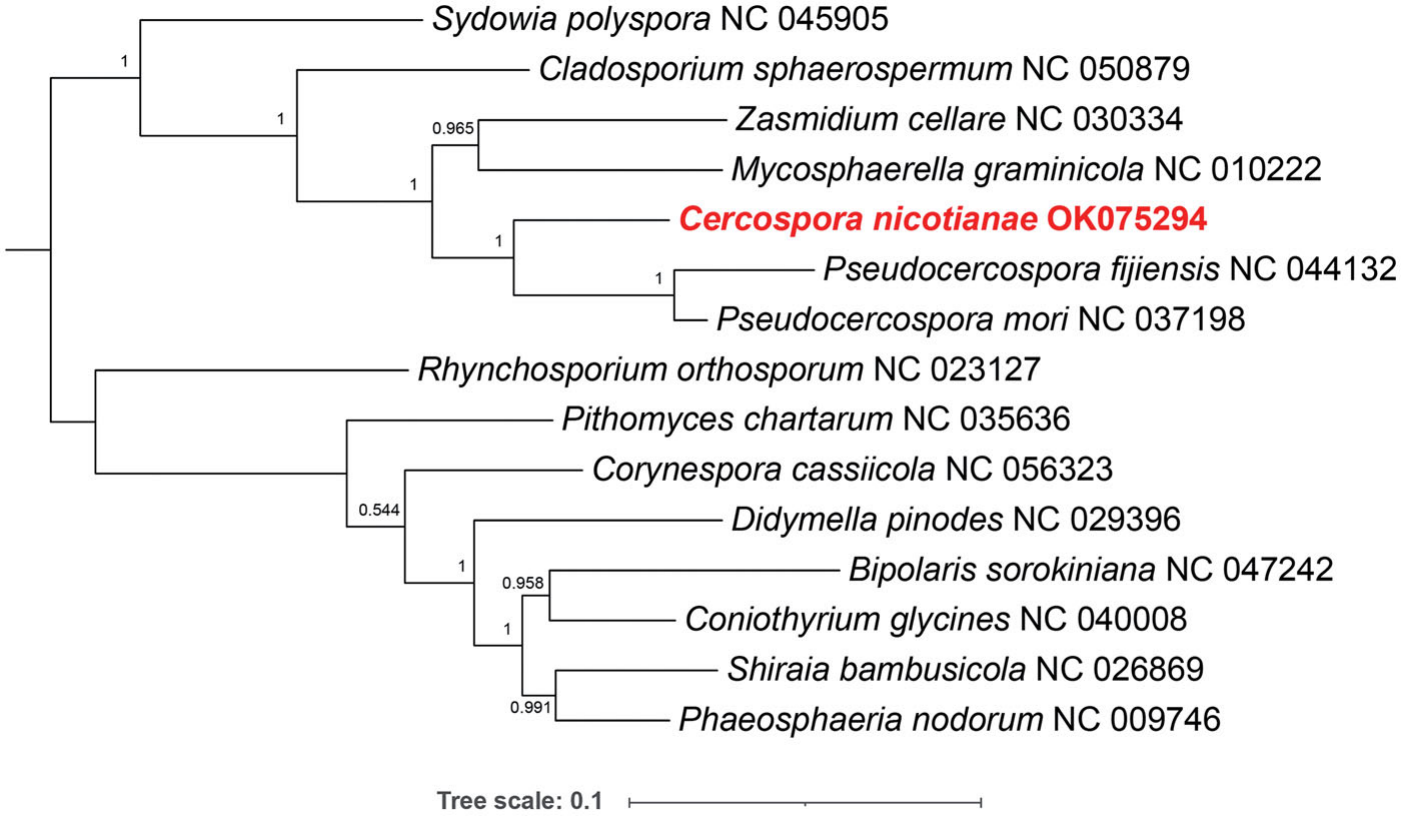
Phylogenetic relationship of 15 species constructed by the Maximum Likelihood method based on the 12 core protein-coding genes.

## Data Availability

The genome sequence data that support the findings of this study are openly available in GenBank of NCBI at (https://www.ncbi.nlm.nih.gov/nuccore/OK075294.1) under the accession no OK075294. The associated BioProject, SRA, and Bio-Sample numbers are PRJNA770472, SRR16555325, and SAMN22220407 respectively.
